# Text Mining Mental Health Reports for Issues Impacting Today’s College Students: Qualitative Study

**DOI:** 10.2196/10032

**Published:** 2018-10-23

**Authors:** Fay Cobb Payton, Lynette Kvasny Yarger, Anthony Thomas Pinter

**Affiliations:** 1 Poole College of Management North Carolina State University Raleigh, NC United States; 2 College of Information Sciences and Technology Pennsylvania State University University Park, PA United States; 3 Department of Information Science University of Colorado Boulder Boulder, CO United States

**Keywords:** text mining, mental health, college students, information and communication technologies

## Abstract

**Background:**

A growing number of college students are experiencing personal circumstances or encountering situations that feel overwhelming and negatively affect their academic studies and other aspects of life on campus. To meet this growing demand for counseling services, US colleges and universities are offering a growing variety of mental health services that provide support and services to students in distress.

**Objective:**

In this study, we explore mental health issues impacting college students using a corpus of news articles, foundation reports, and media stories. Mental health concerns within this population have been on the rise. Uncovering the most salient themes articulated in current news and literature reports can better enable higher education institutions to provide health services to its students.

**Methods:**

We used SAS Text Miner to analyze 165 references that were published from 2010 to 2015 and focused on mental health among college students. Key clusters were identified to reveal the themes that were most significant to the topic.

**Results:**

The final cluster analysis yielded six themes in students’ mental health experiences in higher education (ie, age, race, crime, student services, aftermath, victim). Two themes, increasing demand for student services provided by campus counseling centers (113/165, 68.5%) and the increased mental health risks faced by racial and ethnic minorities (30/165, 18.2%), dominated the discourse.

**Conclusions:**

Higher education institutions are actively engaged in extending mental health services and offering targeted outreach to students of color. Cluster analysis identified that institutions are devoting more and innovative resources in response to the growing number students who experience mental health concerns. However, there is a need to focus on proactive approaches to mitigate the causes of mental health and the aftermath of a negative experience, particularly violence and sexual assault. Such strategies can potentially influence how students navigate their health information seeking and how information and communication technologies, including mobile apps, can partially address the needs of college students.

## Introduction

While attending college, students may experience personal circumstances or encounter situations that feel overwhelming and negatively affect their academic studies and other aspects of life on campus. To meet this growing demand for counseling services, US colleges and universities are offering a growing variety of mental health services that provide advice, counseling, and services to students in distress. For example, in 2015, the Center for Collegiate Mental Health delivered their annual report of data contributed by member institutions. There were 100,736 unique college students seeking mental health treatment, 2770 clinicians, and 770,000 appointments across 139 college and university counseling centers [1]. The following year, the center reported that there were 150,483 unique college students seeking mental health treatment (a 50% increase from the previous year) from 3419 clinicians. This resulted in over 1 million appointments across the 139 college campuses [2]. Half the students seeking counseling were already in some form of treatment, and one-third were prescribed medication before their arrival to campus. In 2017, the center reported that there were 161,014 unique students seeking treatment, 3592 clinicians, and 1,255,052 appointments across 147 institutions. When compared with the data presented in the 2015 report, this represented a 60% increase in unique students seeking help and a 62.9% increase in appointments requested, whereas only a 29.6% increase in available clinicians.

The National Alliance on Mental Illness (NAMI) 2012 report entitled *College Students Speak: A Survey Report on Mental Health* is based on a national survey of 765 college students diagnosed with a mental health condition [3]. The sample was predominantly female (627/765, 82%), with fewer male (122/765, 16%) and transsexual (15/765, 2%) respondents. In all, 82% (627/765) of the respondents were white. African Americans (46/765) and multiracial students (46/765) equally represented 6% of the sample, whereas Asian Americans (38/765) and Hispanics/Latinos (38/765) accounted for equal representation at 5% each. Another 3% (23/765) of the sample was American Indian, and 1% (8/765) were Pacific Islanders. Students noted various concerns regarding mental health on college campus—namely, dropping out of school, lack of disclosure (and the associated stigma), necessity of awareness activities, deficiencies in online information-seeking skills, appropriate avenues for securing academic accommodations, and clinical and crisis support. Despite these needs for mental health resources and counseling services, health information- and help-seeking practices vary widely among young adults with regard to race/ethnicity, content type, content developer, and culture along with social and traditional media platforms [4-8]. According to Substance Abuse and Mental Health Services Administration (SAMHSA), persons between the ages of 16 and 25 years with a mental health condition are less likely than other age groups to seek and receive health information and assistance [9].

A *New York Times* article reported how higher education institutions are addressing depression and mental illness among college students and implementing outreach efforts to increase both awareness and availability of health services [10]. Regardless of age, mental health has significant economic and social implications that impact daily living. Salient to college students, mental health issues are associated with lower grade point averages, higher probability of dropout rates, and increased stigma [3,8,10,11].

In October 2016, *The Chronicle of Higher Education* released its compilation, *Mental Health Issues in Students* [12]. This report is a collection of nine articles discussing the growing trend of increased mental health stressors experienced by college students and details initiatives that several colleges are using to address these issues. The report stated an alarming statistic: “Nearly one-third of university counseling centers must put students seeking help on waiting lists” (p 3). Moreover, limited financial resources among some students as well as constrained, and even shrinking, institutional budgets continue to point to the need to uncover the major themes associated with the mental health discourse.

While a growing number of US citizens are using online sources for health information seeking [13], college students use the internet more often than the wider population. There is widespread use of cellular phones and other mobile devices, computers, Web-based technologies, and social media for accessing and disseminating information. However, there is a substantial need for mobile health services for youths and young adults [14], but some evidence-based and empirically validated research is taking place. For example, researchers developed a moderated online social therapy intervention (MOST+) to deliver anonymous evidenced-based mental health care in real time. The tool provides Web chat counseling with a clinician, interactive user-directed online therapy, expert and peer moderation, and private and secure peer-to-peer social networking [14]. Others examined mental health support for African American college students (who are less likely to seek mental health support from a university institutional service) and reported that microblogging (eg, Tumblr) can be a powerful tool for expressive and instrumental social support. In addition, user-centered and participatory design of these information and communication technology (ICT) platforms can offer health care professionals, educators, and providers insights into how to better engage target populations, improve medical decision making, and increase efficacy in the technology design process [15,16].

Given the increased attention to mental health among college students, the purpose of our study is to uncover hidden patterns and themes in news articles, foundation reports, and media stories on our topic to identify areas that have garnered attention as well as areas that have been underexplored. To better understand these dynamics, we collected and analyzed via text mining methods news articles, foundation reports, and media stories related to mental health and college students. Our study applies a text mining approach discussed by Ananiadou and colleagues [17] for accelerating the process of systematic analysis for the domain of collegiate mental health. We contend that using an inductive approach to uncovering these factors can provide insights to academic institutions and mental health service providers as they seek to improve awareness and create culturally relevant services and activities as well as cultivate climates that are supportive and inclusive of and responsive to diverse student populations. These institutional efforts are particularly critical given recent college campus debates around social and political activism, the microaggressions that ensue, sinking budgets, and increased legal and ethical conflicts confronting higher education.

## Methods

### Data Source

Text mining can be defined as the computational discovery of new, previously unknown information by automatically extracting information from different written sources [18]. Text mining combines computational and statistical methods to extract previously unknown information from heterogeneous and unstructured written documents. Text mining draws from a number of fields, including data mining, machine learning, natural language processing, computational linguistics, statistics, and information retrieval [19].

The text mining process starts by curating a large collection of documents, which can come from disjointed and disparate literature. The documents can include unstructured and semistructured data, such as email and full-text documents. The curation process can be done manually by researchers, but this laborious task requires considerable expertise [20]. When automated, text mining can greatly reduce curation time and produce a larger corpus of news articles, foundation reports, and media stories. Although the overall accuracy of the corpus may be lower [20], analyzing data from heterogeneous sources is a common problem in data mining applications. However, work from other fields, such as biology, biomedicine, and oncology, has found utility in analyzing heterogeneous data sources in tandem [21].

After the curation process, whether a hands-on literature search or computationally produced, the text mining tool will retrieve a particular document and preprocess it by checking format and character sets. Finally, in the text analysis phase, techniques such as clustering, categorization, concept linkage, topic tracking, and information visualization are used to discover information embedded in the documents [19]. Text analysis facilitates the extraction of information from the increasing body of text online in scholarly, open access publications.

Researchers [22] discussed the role of text mining in health care contexts, offering that, “Text mining is of interest because a large volume of ‘unstructured’ data (eg, narratives, event descriptions) is submitted as part of adverse event reporting” (p 429).

Applying text mining methods to mental health narratives, we identified articles including both college students and mental health published from 2010 to 2015 in *The Chronicle of Higher Education*, *Insider Higher Education*, *Diverse*, *Ethnic Newswatch,* and the *Journal of Blacks in Higher Education*. In total, we collected 165 documents using combinations of the keywords, “college students,” “mental health,” and “mental services.” This collection was comprised of two parts: (1) identifying potential sources and keywords and (2) searching sources using the keywords and saving articles that met a set of criteria and formed our corpus of heterogeneous data sources [21]. Duplicate articles were removed from the corpus to avoid overcounting any member of the corpus or biasing the data.

We aggregated 165 articles that we analyzed in SAS Enterprise Miner with Text Miner 12.1. SAS Text Miner produces four outputs: (1) a text parsing analysis that pulls words from the documents and determines its part of speech and importance, (2) a text filtering analysis that determines which words to discard or keep for further analysis, (3) a text topic analysis that groups articles into similar topics defined by words, and (4) a text cluster analysis that places articles into clusters based on common words that are shared between articles. These steps in our quantitative analyses are automated using SAS Text Mining. In a secondary portion of our analyses, we adopted a qualitative approach using quotes from the corpus to engage in a sensemaking process (acquisition, reflection, and action) to better understand context and meaning [23].

## Results

### Quantitative Results

#### Parsing and Filtering

**Figure 1:**
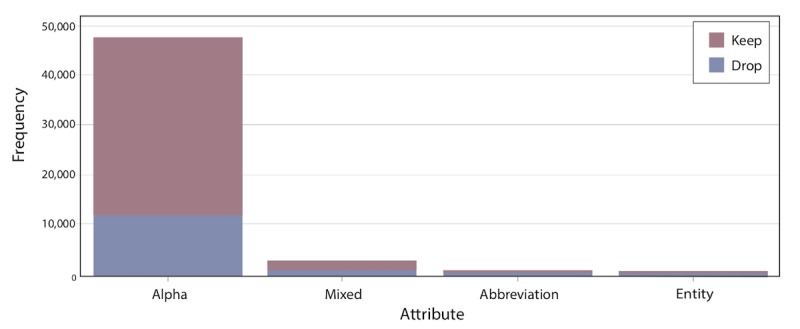
Text filtering results: attributes by frequency.

**Figure 2:**
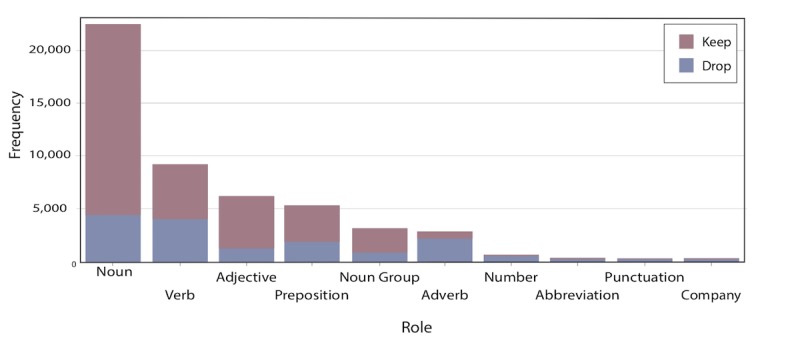
Parts of speech from text filtering: roles by frequency.

The first two steps in the text mining process are parsing (Table 1) and filtering (Figures 1 and 2). These two steps enabled us to identify the initial role (eg, noun, verb, adjective) and attribute (eg, alpha, mixed, abbreviation, entity) of each term in the corpus, and remove insignificant terms (eg, the, a, an, be, do) from the parse. In the parsing analysis, the attributes refer to the importance of the word. Abbreviations and entities are unimportant, mixed may be important, and alpha is important.

#### Text Topic

The text topic analysis identified a total of 25 topics; however, three were concerned with sharing the articles themselves or with other extraneous data. Therefore, there were only 22 salient topics identified by the text topic analysis. In Table 2, we present all 25 topics (the three removed topics are ID numbers 23, 24, and 25). Table 2 also contains the number of terms present in the dataset and the number of documents represented in each topic.

Table 2 illustrates themes regarding college-lived experiences related to current events of civil unrest on campuses. For instance, there are substantial terms and documents containing words such as “Virginia, racism, Berkeley, tweet, college services.” These are undoubtedly stressful campus activities related to recent social and cultural events (eg, University of Virginia, Berkeley, violence, protests, counterprotests, racism, racial), use of social media technologies (eg, Facebook, Twitter), college matriculation (eg, graduate student, institution), and mental health concerns (eg, well-being, depression, sexual assault, and accommodation).

**Table 1:** Text parsing results: roles and attributes by frequency.

Term	Role	Attribute	Frequency	Documents (N)	Keep	Status	Weight
Be	Verb	Alpha	11,484	136	No	Drop	0.000
A	Noun	Alpha	5672	136	No	Drop	0.000
Student	Noun	Alpha	5065	136	Yes	Keep	0.183
Other	Adjective	Alpha	1139	131	No	Drop	0.000
University	Proposition	Alpha	1483	131	Yes	Keep	0.180
Campus	Noun	Alpha	1454	119	Yes	Keep	0.179

**Table 2:** Text topic analysis results.

Topic ID	Topic	Terms (n)	Documents (n)
1	commission, reform, virginia, commitment, involuntary	691	4165
2	stalk, violence, crime, domestic, domestic violence	353	12,165
3	block, open, abstract, newspaper article, scholunivauthors	519	8165
4	foundation, poll, kids, Harris, en	537	6165
5	Penn, ic, Berkeley, stu, Tucker	1013	9165
6	receipt, health, black, care, health	545	16,165
7	grad link, graduate, graduate student, Facebook	755	19,165
8	food, insecurity, food homeless, hungry	491	9165
9	Montclair, minority, program outreach, home	411	7165
10	duty, UCLA, foreseeable, court, protect	521	9165
11	counsel, counsel center, center, link, institution	566	24,165
12	cortisol, depressive, disorder, warning, alcohol	796	11,165
13	fund, Steve, Evan, line, rose	560	10,165
14	fax, phone, yes, north, institutions	771	4165
15	racism, black student, black, racial, color	678	18,165
16	institution, service, student, program	765	16,165
17	gun, conceal, law, weapon, ban	543	12,165
18	mental health, mental health condition, accommodation	586	18,165
19	harassment, academy, sexual harassment, sexual, assault	586	11,165
20	percent, student, sexual, report, college	874	11,165
21	brain, DSM, illness, mental, NIMH	726	14,165
22	institution, mental, college, health, athlete	708	17,165
23	well-being, scale, sample, item	698	10,165
24	link, Facebook, Twitter, share, APA	772	14,165
25	user, tweet, PTSD, et al, de	827	9165

#### Text Cluster

The text cluster analysis identified six distinct clusters of articles in the dataset. In Table 3, we present each of the clusters, a brief description of the cluster based on the keywords that SAS produced, and document frequency and percentage of sample statistics. Although there were six identified clusters, these clusters could be further grouped into two broad themes: (1) factors that play a role in the occurrence of mental health experiences in higher education (clusters 1, 2, 3, and 6) and (2) what happens after a mental health experience (clusters 4 and 5). Table 3 depicts these clusters and shows an emphasis on mental health services followed by race as the major themes in the data.

### Qualitative Results

#### Factors in Mental Health Experience

As shown in Table 3, the cluster analysis identified six main foci when discussing themes in mental health experience in higher education. We sought to better understand the clusters via sensemaking from the set of documents linked to the SAS Text Miner results. The quotes provided subsequently are derived from the documents that appeared in the associated clusters rather than from our mere selection of data/quotes from the corpus.

Cluster 1 focused on the ways that age plays in a role in mental health experiences in the university environment. The literature in this cluster is primarily focused on younger students and the challenges they experience in the transition from high school to college and during their first year of higher education. Students reported that college preparation focused on academic preparation, often leaving emotional preparation by the wayside. Yet, emotional preparedness was correlated to academic success (“Students who feel...”). The lack of emotional preparation often led to an increase in stress and mental health experiences [24] that colleges are not prepared to handle as discussed in *The American Freshman: National Norms* [25].

**Table 3:** Cluster analysis results.

Cluster	Name	Description	n (%)
1	Age	Age factors in mental health experiences	7 (4)
2	Race	Racial factors in mental health experiences	30 (18)
3	Crime	Factors related to crime that lead to mental health experiences	3 (2)
4	Services	What institutions are doing to assist with mental health experiences	113 (68)
5	Aftermath	What happens after a mental health experience	4 (2)
6	Violence and sexual assault	Factors that can lead to mental health experiences	8 (5)

Freshmen often relied on friends, family members, or substance abuse to cope with the stress of the transition from high school to college [25]. Prior research has demonstrated that substance abuse is a contributing factor to further negative mental health experiences [3,24,26], and students without familial support networks might be disadvantaged by the lack of institutional resources, leading to further negative outcomes.

Extant work demonstrated a connection between race and mental health experiences (cluster 2)—namely, minority groups are more likely to have difficulty with the high school to college transition and with the higher education environments as a whole. Black students were shown to feel less emotionally prepared than their white counterparts [7] leading to academic, social, and cultural struggles characterized by minimal diversity and exclusivity [27].

Moreover, although black students are less likely to engage in alcohol consumption, research found that black students were less likely to seek help and act on mental health support or they received less effective support than majority students [24,28], particularly at predominantly white institutions [29]. As Terri Wright, the executive director of Steve Fund, which assists colleges in improving their mental health services for students of color, informed National Public Radio [30], “Stigma is a huge issue in the lives of students of color and what it means to seek services and admit that I need help, when in fact I feel like, as a young person of color, that I’m already being judged differently.”

Cluster 4 (services) emerged as the largest topic found in the corpus. Per the NAMI *College Students Speak* report [3], appropriate and multiprong delivery of mental health services is essential on today’s university campuses. Health information seeking on the part of students and roles of delivery by counseling centers, in part, influences how services are accessed and provided. The Association for University and College Counseling Center Directors recognizes the revolving needs for mental health services and the broad experiences that students report. Textbox 1 provides the positive and negative experiences associated with on-campus health services per the student participants in the NAMI report [3]. A careful examination of Textbox 1 does not include topics of diversity of services, counselors themselves, location (rural versus urban), and cultural differences, and as researchers [31] concluded in an analysis of the 2007 Virginia Tech tragedy, the need to “connect the dots” among campus stakeholders and faculty, campus police, and counseling center staff.

Higher education institutions also acknowledge that they are not prepared to deal with students’ needs but are attempting to rectify this in various ways. Community colleges, for example, are turning to local partners to outsource the need for mental health services. Some universities are building partnerships with local care providers to bridge the gaps in their support networks. Others are utilizing teletherapy and mobile phone apps to target students in need, databases to track students’ health needs, and self-assessment tools that enable institutions to identify areas for improvement in mental health care services. By 2014, 55 colleges had partnered with the Jed Foundation and Clinton Foundation for a review of their mental health services focused on substance abuse and suicide prevention [32].

From these clusters, it is clear that merely being in college has an impact on the mental health of students, and that there are disparities in the level of these experiences and how universities deliver care to those affected. In addition to these demographic factors (eg, age, race) and the looming needs for additional services, there were external factors that the analysis identified as contributing to mental health experience in the university setting. These were violence and sexual assault (cluster 6) and crime (cluster 3) experiences that students experience while in college.

Although clusters 1 to 4 can be seen as validating what the field has previously reported, clusters 5 and 6 were unanticipated. The findings are absent of any discussion related to institutional staff. Although staff are absent from our findings, they are critical to the delivery and assessment of mental health services on university campuses. Staff are continually in need of mental health training, and this is in light of shrinking institutional budgets [12]. In addition to and despite privacy concerns, more counseling centers are implementing technology applications to assist with increase caseloads and seeking to find effective ways to provide mental health services [12]. Lastly, the increased need for student mental health services is far outpacing the rate of staff and expertise needed to address these issues.

The literature dealing with violence and sexual assault focused primarily on new legal requirements for universities and colleges to compile and report annual statistics about it in the higher education environment. Other articles discussed how to handle these incidents in compliance with federal Title IX regulations. There was no discussion of how to prevent these incidents from occurring or the effects they may have on victims, which is interesting given that research has demonstrated a link between sexual assault and negative mental health experiences [33].

**Students’ evaluation of mental health services and supports from the National Alliance on Mental Illness (2012) [2].**

Top five reasons students found services and supports good

1. There is effective coordination between students, treatment providers, professors, and the Disability Resource Center (DRC).
2. There is free group and individual counseling offered on campus.
3. There is variety and flexibility. Help is available 24 hours a day, seven days a week.
4. There are permanent, qualified, and caring mental health staff members, including on-site psychiatrists.
5. There are supportive students, faculty, and staff who participate in mental health groups and training.

Top five reasons students found services and supports poor

1. There are a limited number of counseling visits allowed on campus and a limited number of resources.
2. There are not enough adequately trained mental health providers.
3. The college does not recognize the importance of peer support.
4. There is a lack of communication between mental health providers and others involved in students’ care.
5. The college is too quick to prescribe medications or hospitalize students with mental health issues.

What literature did touch on was that universities do not have a legal obligation to protect adult students from third parties. In a California Court of Appeals ruling, it ruled that the University of California system could not be held liable for the attack of a student by a mentally ill student. The dichotomy between these two clusters illustrates a disconnect that exists between theory and practice. Although institutions of higher learning are required to report statistics of violence and sexual assault, they are not required to prevent individuals who might be classified as at-risk from accessing campuses. This points to a clear need to identify gaps in community services, including crisis intervention and case management services [31].

The clusters in this theme demonstrated that higher education is woefully underprepared to assist those who undergo negative mental health experiences. High schools and colleges prepare their students for academic—not emotional—success, leaving young and minority students in danger of suffering negative health outcomes. They also emphasize reporting the prevalence of attacks that might be caused by or cause mental health outcomes but are not required to act on these instances. With the absence of laws and regulations requiring action, it is left to the individual institution to act in a way to benefit their students and minimize risk to the higher education community at their campuses. This lack of standardization leads to an uneven approach to mental health that varies by university; some universities are proactive about their students’ well-being, whereas others simply fulfill the legal requirements. This leads to the aftermath theme (cluster 5) observed in our cluster analysis.

The high number of topics identified stands in stark contrast to the six clusters identified in the literature. This highlights that the discussion of mental health in higher education is a nuanced conversation—each case is different. It is limiting to distil the mental health conversation into six clusters. Instead, the discussions and investigations that researchers undertake should reflect these nuances. Although cluster analysis identified that institutions are devoting resources to help students who experience mental health issues, it identified far fewer instances of understanding how to mitigate the causes of mental health.

## Discussion

### Principal Findings

The final cluster analysis yielded six themes in students’ mental health experiences in higher education (ie, age, race, crime, student services, aftermath, victim). These clusters were grouped into two broad categories: (1) factors that play a role in the occurrence of mental health experiences in higher education (age, race, crime, victim) and (2) factors describing what happens after a mental health experience (student services, aftermath). Our data analyses show student services and race are the primary themes in the literature, whereas violence and sexual assault are lesser-covered topics.

### Recommendations for Colleges and Universities

The text topic node identified a variety of stressors and traumatizing experiences, such as sexual assault, food scarcity, violence, and racism, that lead students to seek rapid-access mental health support and services. The Center for Collegiate Mental Health [2] reports that participating counseling centers, on average, are providing 28% more “rapid-access” service hours per client but 7.6% fewer “routine” service hours per client over the period from 2010 through 2016. This may suggest a void in the provision of follow-up routine care for students who receive rapid care, university funding models that do not increase treatment capacity, as well as clients terminating their routine care (eg, no shows, appointment cancelations, end of the academic term) before their treatment goals are achieved. We call on higher education institutions to focus on more proactive approaches to mitigate the stressors that can cause mental health episodes among students. A recommendation for moving forward would be to focus on helping students to develop coping skills before they experience a traumatizing event, instead of primarily focusing on the postexperience treatment. In the long run, this proactive shift could enable institutions to reduce demand for rapid-access services, alleviate their overworked mental health practitioners, and lead to a better higher education experience for students.

In addition, unanswered issues remain, namely (1) what happens after a negative mental health occurrence on campus? and (2) what are higher education institutions doing to assist their communities in coping with these experiences? There were several large themes that we observed comprising these clusters. These included subsets of literature that discussed faculty’s role, stigma associated with mental health services, how institutions attempt to help students through tracking mechanisms, technology apps, and counseling centers, and how they are being funded and utilized [12]. Legal and ethical conflicts, however, continue to pose challenges to how higher education does and will approach these unresolved issues.

Literature examining faculty’s role in helping students who experience mental health events was split between two views: (1) faculty should be active in assisting students and (2) faculty involvement should be limited due to a lack of training which might do more harm than good to students in an intervention. This split illustrates the institution-by-institution approach to mental health that the higher education system takes; hence, leaving some universities unprepared or underprepared to deal with their students’ mental health needs. Although some institutions actively fight for better mental health care and individual faculty members weigh the pros and cons of disclosing their own personal mental health issues [34], some institutions instead seek to limit students’ abilities to opt out unless they have a valid medical reason [30]. However, student governments are increasingly advocating for mental health awareness [35] while groups, such as Active Minds [36], are pushing for national reform of the university mental health care services [37]. University administrators, alumni, students, and other stakeholders are recognizing the dramatic increase in utilization of counseling services and are establishing endowments to support mental health services [38]. Students are also addressing the need for better safety nets for young college students and for students who develop serious mental health issues while in college. When a student body comes together, it might frequently expedite a university’s response to an issue [39-41]. Lastly, the role of staff is another vital factor that emerged from our work.

### Recommendations for ICT Interventions

Our study also offers suggestions for mobile interventions. Prior research has found that individuals with serious mental illnesses own mobile devices at lower rates than the general population. However, despite mobile device ownership or nonownership, people desire mobile services to help cope with their illness [42,43]. In particular, African Americans and Hispanics are using mobile phones to access health information via the internet more frequently than those who classify themselves as white [44]. The higher prevalence of mobile phone use among blacks, along with our finding that race is the dominant theme in discourses about mental health issues on US college campuses, provides unique opportunities for those in public health research and health education to reach these historically underserved populations using mobile health interventions [44].

Our study highlights salient mental health issues for researchers seeking to develop impactful mobile interventions. Additional evidence-based research is needed in this domain. Prior research has identified five existing apps that targeted depression, anxiety, and substance abuse, but noted that these were the only apps that relied on evidence-based research [45]. Researchers went on to note that a majority of commercially available apps do not have scientific evidence backing their efficacy, leaving a gap for research to address with regards to creating better mental health apps.

Although ICT such as teletherapy and mobile phone apps has not yet been the magic bullet to cure health conditions and chronic diseases, it is a promising tool in the field of health promotion and literacy particularly for college-aged technology users. Researchers are reporting health gains in their assessments of mental health mobile apps [46]. For example, Miyamoto and colleagues [45] conducted focus groups with 30 adults and found that mobile health apps can be used to track health data and encourage sustained behavior changes to support health goals. Andersen and colleagues [44] reviewed the scholarly literature and found that mobile apps have the ability to increase prevention and health education in health-disparate communities. Thom et al [47] studied depression, anxiety, and internet use among US teens and found that internet use may mitigate anxiety in adolescents with higher levels of baseline anxiety.

Researchers [48-50] note that mobile technology use supports mental well-being both as an information resource and as a tool for providing interventions and treatments. Mobile apps are used and developed for symptom assessment, psychoeducation, resource location, and tracking of treatment progress. Mobile apps can enable patients, caregivers, and clinicians to assess treatment, mood, stress, anxiety, and location via global positioning system (GPS) tracking. Evidence-based treatments, such as digital diaries, text messaging, video and audio captures, and virtual training for therapeutic skills, are capabilities supported by current mobile apps. Moreover, the mental health mobile apps and digital storytelling can be powerful tools to encourage college students and others to discuss their experiences, provide emotional support, and allow young people mechanisms to explore and artfully share their own stories and thoughts [51].

According to a 2013 survey by the Association for University and College Counseling Center Directors, nearly 6% of the 380 colleges participating in the study now use teletherapy. Although that number might not seem high, it is up from less than 0.5% in 2012. However, legal issues abound for therapists, including college counselors. Current laws require therapists to be licensed in the state in which they practice, so they may not be able to provide mental health services to students in different states. That is especially relevant to students who may be in a different state or enrolled in distance education [37].

### Limitations

Although this work evaluates mental health among college students via text mining mainstream reports, articles, and academic sources, it is not without limitations. Text analysis is a descriptive method which informs us what but not why—hence underlying patterns are not revealed. Secondly, the analysis is limited by the content used in the corpus. More experimentation via other text mining terms and other databases could provide additional insights to our findings. We use a text mining approach to analyze the reports and writings, but this study does not focus specifically on how technology can be used to address the issues of mental health among college students. Third, the professoriate is challenged to uncover and address the needs of its students holistically. This, in part, requires attention to who is impacted and unearthing their stories. As reported in *The Chronicle of Higher Education* by Quintana [52], MIT professor of computer science, Daniel Jackson, calls mental health’s aftermath at the institution the “giant iceberg of unhappiness.” Our understanding of students’ concerns regarding academic performance, course loads, nontraditional work schedules, food insecurity, family responsibilities, and their mental well-being will paint a holistic picture. The corpus of news articles, foundation reports, and media stories used for this research manuscript do not address the mental health issues using an intersectional lens of clinical technology or student scenarios (context).

## Abbreviations

ICT: information and communication technology
NAMI: National Alliance on Mental Illness
SAMHSA: Substance Abuse and Mental Health Services Administration
